# Carbuncle of the Coronary Ligament and Neighboring Tissues

**Published:** 1894-09

**Authors:** H. McWhinnie

**Affiliations:** Troy, N. Y.


### CARBUNCLE OF THE CORONARY LIGAMENT AND NEIGHBORING TISSUES.

By H. McWhinnie, D.V.S.

While injuries to the feet and neighboring tissues between the carpus and tarsus respectively, are common in every day practice, still I think the above disease deserves more than an ordinary amount of our undivided attention.

Carbuncle, furunculus, or foot rot, as termed by some writers, and which in former years has been attributed to frost bites, cold, wet, etc., should be eradicated from the progressive practitioner's mind. Why it should spend its force inferior to the carpus and tarsus respectively, can be accounted for when we take into consideration the succession of changes that must undergo in those parts exposed to the slush and filth of city streets, lowering their vitality, thus offering less resistance to the circulation to get rid of its poisonous substances through the now already erethematous skin; for I have failed to see a case of this disease where I was unable to trace it to an absorption through the lymphatic system, setting up an excessive degree of constitutional disturbance. That it is due to a blood poison can readily be understood when we notice the rapid changes that undergo in the tissues affected, and if the patient has not received proper treatment purulent abscesses rapidly form, which tends to take on a gangrenous condition, with rapid sloughing of tissue, producing open joint, or an entire detachment of the hoof, the animal dying in a few hours from septicæmia.

The symptoms are diagnostic of great pain ; for it is a true fact, if we are called to see the patient within six hours after attack we invariably find him with hurried respiration, accelerated pulse, and a temperature averaging from 103 to 106 degrees F., and sweat bedewing the body, with lameness of a severe character, at first without swelling, but in the course of from six to ten hours the surrounding tissue becomes infiltrated with an unhealthy serum which rapidly disintegrates, points, bursts, and discharges a very fetid pus, leaving behind ulcers that tend to coalesce, unhealthy granulating wounds, indolent to heal, and prone to form exuberant granulations.

As to treatment, I am convinced that any application which tends to favor superficial circulation, or the arrest of leucocytes in their course, as hot applications and poultices, are injurious. Therefore I would recommend the treatment I pursue, and in 99 per cent. of my cases prove successful. Keep the limb affected in a pail of ice water for a half hour at a time, five or six times daily. Each time, after bathing, saturate the part with the following lotion:

Plumbi acetatis, 1 oz.

Zinci sulph., 1 oz.

Tr. arnicæ, 1 oz.

Acidi carb. sol., 1 oz.

Aquæ puræ, q. s., 16 oz.

Internally I prescribe one grain of strychnine hypodermically, once daily, for in all cases of blood poisoning I find the vasomotor system of nerves are in a semi-paralyzed condition, necessarily causing a sluggish circulation, and thereby delaying a rapid elimination of excrementitious substances from the system. This, together with one ounce of Fowler's solution and two drachms of the Tr. of Ferri Mur. daily, constitutes my treatment. The fever I pay no attention to unless it becomes alarmingly high. Treat the biood entirely; remove the cause and the effects will cease.

Troy, N. Y.

